# Native T1 mapping: inter-study, inter-observer and inter-center reproducibility in hemodialysis patients

**DOI:** 10.1186/s12968-017-0337-7

**Published:** 2017-02-27

**Authors:** Matthew P. M. Graham-Brown, Elaine Rutherford, E. Levelt, Daniel S. March, Darren R. Churchward, David J. Stensel, Christie McComb, Kenneth Mangion, Samantha Cockburn, Colin Berry, James C. Moon, Patrick B. Mark, James O. Burton, Gerry P. McCann

**Affiliations:** 1 John Walls Renal Unit, University Hospitals Leicester NHS Trust, Leicester, UK; 2 Department of Infection Immunity and Inflammation, School of Medicine and Biological Sciences, University of Leicester, Leicester, LE1 9HN UK; 3 National Centre for Sport and Exercise Medicine, School of Sport, Exercise and Health Sciences, Loughborough University, Loughborough, UK; 4 BHF Cardiovascular Research Centre, Institute of Cardiovascular and Medical Sciences, University of Glasgow, 126 University Place, Glasgow, UK; 5 The Glasgow Renal & Transplant Unit, Queen Elizabeth University Hospital, 1345 Govan Road, Glasgow, UK; 6 Department of Cardiovascular Sciences, University of Leicester and NIHR Leicester Cardiovascular Biomedical Research Unit, Glenfield Hospital Leicester, Leicester, UK; 7 Clinical Physics, NHS Greater Glasgow and Clyde, Glasgow, UK; 8 West of Scotland Heart and Lung Centre, Golden Jubilee National Hospital, Clydebank, UK; 9 UCL Institute of Cardiovascular Science, University College London, London, UK

**Keywords:** Hemodialysis, Myocardial fibrosis, Native T1, Reproducibility, Cardiovascular magnetic resonance

## Abstract

**Background:**

Native T1 mapping is a cardiovascular magnetic resonance (CMR) technique that associates with markers of fibrosis and strain in hemodialysis patients. The reproducibility of T1 mapping in hemodialysis patients, prone to changes in fluid status, is unknown. Accurate quantification of myocardial fibrosis in this population has prognostic potential.

**Methods:**

Using 3 Tesla CMR, we report the results of 1) the inter-study, inter-observer and intra-observer reproducibility of native T1 mapping in 10 hemodialysis patients; 2) inter-study reproducibility of left ventricular (LV) structure and function in 10 hemodialysis patients; 3) the agreement of native T1 map and native T1 phantom analyses between two centres in 20 hemodialysis patients; 4) the effect of changes in markers of fluid status on native T1 values in 10 hemodialysis patients.

**Results:**

Inter-study, inter-observer and intra-observer variability of native T1 mapping were excellent with co-efficients of variation (CoV) of 0.7, 0.3 and 0.4% respectively. Inter-study CoV for LV structure and function were: LV mass = 1%; ejection fraction = 1.1%; LV end-diastolic volume = 5.2%; LV end-systolic volume = 5.6%. Inter-centre variability of analysis techniques were excellent with CoV for basal and mid-native T1 slices between 0.8–1.2%. Phantom analyses showed comparable native T1 times between centres, despite different scanners and acquisition sequences (centre 1: 1192.7 ± 7.5 ms, centre 2: 1205.5 ± 5 ms). For the 10 patients who underwent inter-study testing, change in body weight (Δweight) between scans correlated with change in LV end-diastolic volume (ΔLVEDV) (*r* = 0.682;*P* = 0.03) representing altered fluid status between scans. There were no correlations between change in native T1 between scans (ΔT1) and ΔLVEDV or Δweight (*P* > 0.6). Linear regression confirmed ΔT1 was unaffected by ΔLVEDV or Δweight (*P* > 0.59).

**Conclusions:**

Myocardial native T1 is reproducible in HD patients and unaffected by changes in fluid status at the levels we observed. Native T1 mapping is a potential imaging biomarker for myocardial fibrosis in patients with end-stage renal disease.

**Electronic supplementary material:**

The online version of this article (doi:10.1186/s12968-017-0337-7) contains supplementary material, which is available to authorized users.

## Background

There is an increased risk of cardiovascular (CV) mortality in chronic kidney disease (CKD), and end stage renal disease (ESRD) populations [1]. This increased risk can be attributed to the stereotyped changes that lead to the development of uremic cardiomyopathy and include, left ventricular (LV) hypertrophy, LV dilatation and myocardial fibrosis within the extracellular matrix [2]. To date, LV mass is the most commonly used surrogate end-point of mortality in clinical trials [3], as observational studies of HD patients have shown LV mass is good predictor of CV outcomes [4]. However, a recent systematic review and meta-analysis in patients of all stages of CKD suggested that there is no clear association between intervention-induced LV mass reduction and mortality [5]. Novel imaging biomarkers that can robustly and reliably measure pathological CV changes that link strongly to outcomes are required. Post-mortem studies of patients with CKD and ESRD on hemodiaysis (HD) demonstrate that uremia is a highly significant, independent determinant of extent of myocardial fibrosis [6]. Furthermore endomyocardial biopsy studies have shown that extent of myocardial fibrosis is the only independent predictor of death (mean follow-up period 3.1 years) for these patents [7]. As degree of myocardial fibrosis is the strongest predictor of increased CV mortality, defining a reliable measure of myocardial fibrosis in HD patients is a priority.

Cardiovascular Magnetic Resonance (CMR) with Late Gadolinium Enhancement (LGE) is an imaging biomarker used to assess myocardial fibrosis in many populations. Whilst gadolinium based contrast agents have previously been used to assess cardiac disease in HD patients [8] this is no longer possible due to the rare, but serious complication of nephrogenic systemic fibrosis [9]. Moreover, whilst LGE is a sensitive and reproducible way of assessing focal myocardial fibrosis, there are limitations in using gadolinium to assess diffuse myocardial fibrosis due to the reliance of the technique on demonstrating a difference between signal intensity of normal and fibrotic myocardial tissue [10]. Native T1 mapping is a novel, non-contrast CMR technique that correlates well with biopsy measured myocardial fibrosis in aortic stenosis [11, 12] and can differentiate patients with hypertrophic cardiomyopathy from hypertensive cardiac disease [13]. The inter-study repeatability and inter-observer variability of native T1 mapping has been shown to be very good in patients with aortic stenosis [14], patients with LV hypertrophy or dilated cardiomyopathy [15] and patients with Anderson-Fabry disease [16]. Myocardial native T1 times have been shown to be significantly higher in HD patients compared to control subjects [17, 18] and to associate with circulating markers of cardiac disease [17] and measures of myocardial systolic strain [18], but the reproducibility of native T1 mapping has not been assessed in HD patients who are prone to shifts in extracellular volume. Native T1 times are prolonged with increasing water content of tissue and the presence of intermittent myocardial edema from alterations in fluid status may in theory affect native T1 time confounding results and reducing native T1 time reproducibility. Concerns remain about the use of this technique to assess myocardial fibrosis in patients with ESRD on HD, who are subject to significant changes in fluid status and who may potentially have intermittent myocardial oedema [19].

In this study we aim to assess the reproducibility and reliability of native T1 mapping in patients with ESRD on HD by assessing: i) the inter-study, inter-observer and intra-observer variability of native T1 times in HD patients at 3 Tesla (3 T); ii) the associations between changes in markers of fluid status and changes in native T1 times to assess the effect of fluid status on native T1 times; iii) the inter-centre reproducibility of native T1 values between two UK cardio-renal imaging centres, comparing analysis techniques of native T1 maps and native T1 values with ‘phantom’ analysis [20].

## Methods

Patients from centre 1 were recruited as a part of the CYCYLE-HD study (ISRCTN 11299707) [21]. The study was given ethical approval by the NHS Research Ethics Committee East Midlands (Northampton; REC ref: 14/EM/1190). Patients from centre 2 were recruited as part of the observational cardiac uraemic fibrosis detection in dialysis patients study (CUDDLE study ISCRTN99591655). The study was approved by the West of Scotland Ethics committee (WoS 13/WS/0301). All participants gave written and informed consent.

### Inter-study reproducibility, inter-observer and intra-observer variability (centre 1)

Ten patients underwent a repeat CMR scan within 2 weeks of their initial scan. Patients receive dialysis three times per week on either a Monday, Wednesday, Friday, or a Tuesday, Thursday, Saturday. This means that there is a two-day break between dialysis once a week, commonly referred to as ‘the long break’. Study patients were all scanned on a non-dialysis day within 24 h after their last dialysis and never during the long-break. The same CMR protocol was used for each scan (see below). We report the inter-study reproducibility of LV volumes, mass and of the mid-ventricular native T1 parametric mapping. Analysis of inter-study scans was conducted by a single, blinded, observer. Inter-observer variability of was conducted by 2 blinded observers on 10 native T1 maps independently from one another. Intra-observer variability was conducted by a single blinded observer.

### Inter-centre reproducibility (centre 1 and centre 2)

The inter-observer variability of native T1 map analysis techniques between 2 UK cardio-renal imaging centres was assessed. The CMR scanners, native T1 mapping sequences and analysis techniques of centre 1 and centre2 were different (see below). Ten basal and 10 mid-ventricular native T1 scans acquired at centre 1 were analysed by a blinded observer at centre 2, using the analysis technique of centre 2 and compared to the results of the analysis of the same scans analysed at centre 1. Conversely 10 basal and mid-ventricular native T1 scans acquired at centre 2 were analysed by a blinded observer at centre 1, using the analysis technique of centre 1 and compared to the results of the analysis of the same scans analysed at centre 2. We also undertook native T1 phantom analysis at both centres. Phantom analyses were undertaken at centre 1 and centre 2 as part of the international, multi-centre T1MES project [20]. Phantom images recorded at a heart rate of approximately 70 beats per minute were obtained and compared. The phantoms were scanned once at each centre and the images were analysed at centre 2. Uniform regions of interest within each relevant phantom area were determined using semi-automated user defined border delineation software (Siemens Argus Analysis Software, Siemens, Erlangen, Germany). The values obtained and their standard deviations were then recorded.

### CMR protocols

The CMR protocols at centres 1 and 2 were pre-defined in the CYCLE-HD and CUDDLE studies respectively. Differences in scan parameters are due to local expertise in gaining the highest quality images possible with the fewest number of artefacts.

### CMR protocol centre 1

All patients were imaged on a 3 T CMR platform (Skyra, Siemens Medical Imaging, Erlangen, Germany) using an 18-channel phased-array anterior coil. Patients were scanned on non-dialysis days, but not after the long-break. The CMR protocols for acquiring cine imaging and native T1 maps were as previously described [18], conforming to internationally recognized standards [22]. Electrographic gated breath-hold steady-state free procession long-axis cine images in 2, 3 and 4 chamber views were acquired. Short axis cine images covering the entire left ventricle were taken at 8 mm slice thickness, 2 mm gap, field of view 300 x 400 mm, matrix 208 x 256, repetition time 2.9 ms, echo time 1.2 ms, flip angle 64-79^0^, temporal resolution <50 ms, with 30 phases per cardiac cycle, in-plane image resolution 1.1 x 1.5 mm to 1.3 x 1.7 mm.

T1 imaging parameters included acquisition of basal and mid T1 maps in 2 LV short-axis slices using the modified look-locker inversion recovery (MOLLI) sequence. Images were acquired using free-breathing with motion correction (MOCO), ECG-gated single-shot MOLLI sequence [23], with 3(3)3(3)5 sampling pattern and the following typical parameters: slice thickness 8.0 mm, field of view 300 × 400 mm, flip angle 50°, minimum TI 120 ms, inversion-time increment 80 ms. MOLLI maps of the left ventricle were acquired at basal and mid-short-axis. The MOLLI sequence was chosen due to the technique’s excellent inter and intra-observer variability at 3 T [24] and because of local expertise [14]. To minimize artefacts, acquisition was performed with the region of interest at isocentre, a small shim volume was applied around the myocardium, a large field of view (400 mm) was used, and imaging was repeated after changing the phase-encoding direction or resonance offset frequency if artefacts persisted.

### CMR protocol centre 2

All participants were scanned on a 3 T CMR platform (Siemens Magnetom PRISMA, Siemens Medical Imaging, Erlangen, Germany) using a 16 channel phased-array anterior coil. As at centre 1 CMR imaging was performed on a non-dialysis day. Image acquisition was ECG-gated and as previously described [17]. T1 imaging parameters included acquisition of basal and mid T1 maps in 2 LV short-axis slices using a MOLLI sequence. Typical acquisition parameters were: slice thickness 6.0 mm, field of view 360 x 307 mm, flip angle 35°, minimum T1 180 ms, inversion-time increment 80 ms, repetition time 267.84 ms, bandwidth 1085 Hertz/pixel.

### CMR scan analysis centre 1

**Fig. 1:**
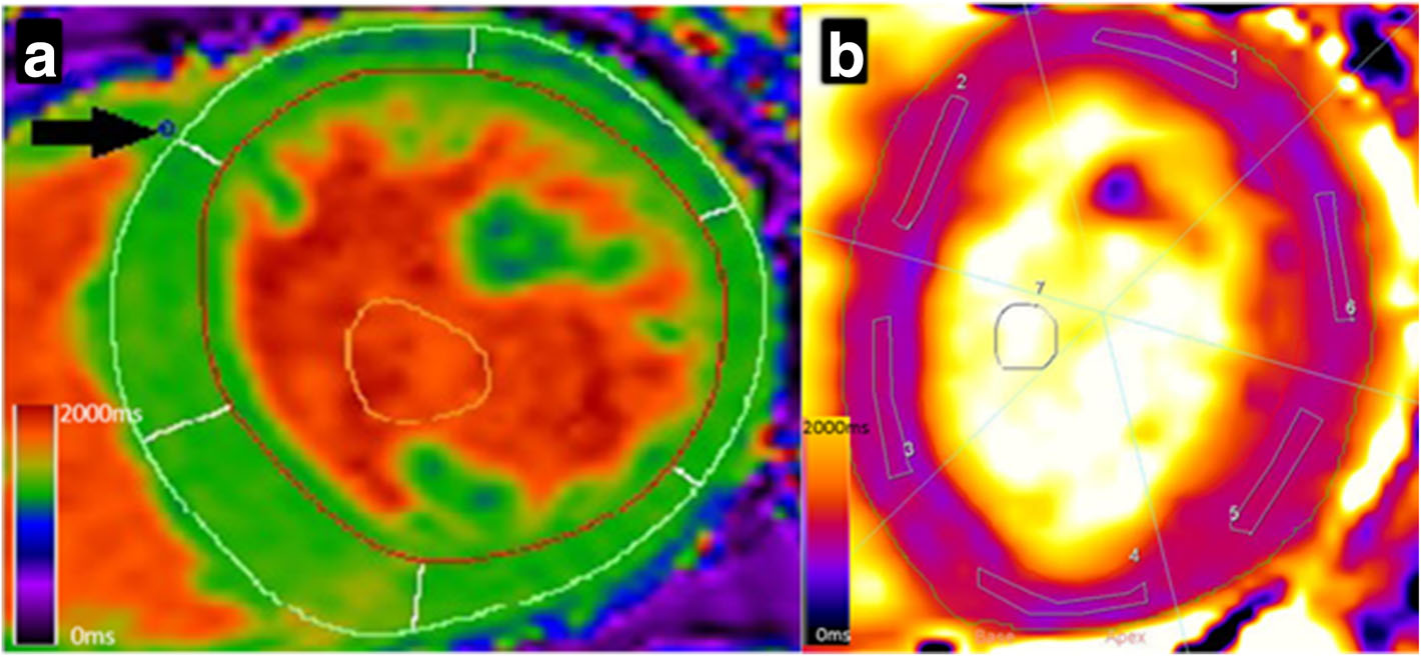
**a**: Native T1 map analysis of a mid-ventricular slice with software package CMR^42^ at centre 1. Endocardial (red line) and epicardial (green line) contouring on native T1 parametric map. Segments (1–6) are calculated from defining the RV insertion point (arrow). **b**: Typical segmentation of a basal native T1 map site from centre 2. 6 discrete regions of interest drawn within the myocardium for each segment

All scans were analysed offline by a single blinded observer using the software package CMR^42^ (Circle Cardiovascular Imaging, Calgary, Alberta, Canada). Image quality was assessed as being excellent, good, acceptable or poor. LV volumes and mass were quantified as previously described with epicardial and endocardial short axis cines at end- diastole and end-systole [25]. The native T1 parametric map derived from MOCO MOLLI images was used to assess native T1 signal due to superior intra and inter-observer variability as described by our group compared to analysing the MOCO series [14]. Using the CMR42 T1 characterization module, endocardial and epicardial borders were drawn on basal and mid-ventricular T1 parametric maps for each patient, with care taken to allow adequate margins of separation from tissue interfaces such as between the blood pool or epicardial fat and myocardium. The anterior right ventricular insertion point was then defined to automatically divide the basal-ventricular and mid-ventricular slices into 6 segments each according to the American Heart Association 16-segment model (Fig. 1). Each individual segment was assessed for the presence or absence of susceptibility and motion artefacts. After removal of any segments affected by artefact, an average T1 time for the whole of the myocardium was calculated from the mean of remaining segments.

### CMR scan analysis centre 2

All scans were analysed offline by a single blinded observer using Siemens Argus Analysis software (Siemens, Erlangen, Germany). The image analysis technique was as previously described [17]. LV contours were defined on raw T1 images (the first image, with the lowest inversion time, allowing for optimal tissue blood-pool contrast) and copied into the parametric T1 maps [26, 27]. Using the anterior right ventricular insertion point as a reference, basal and mid T1 maps were segmented according to the American Heart Association 16-segment model and regions of interest were delineated by user-defined border delineation. The regions of interest were standardized to be of similar size and shape. T1 times were measured in each of the 6 basal and 6 mid segments as well as the blood pool, with care taken to delineate regions of interest with adequate margins of separation from tissue interfaces such as between the blood pool or epicardial fat and myocardium (Fig. 1). Each individual segment was assessed for the presence or absence of susceptibility and motion artefacts. After removal of any segments affected by artefact, an average T1 time for the whole of the myocardium was calculated from the mean of remaining segments.

### Statistical analysis

Statistical analysis were undertaken using SPSS-22 software (Statistical Package for the Social Sciences, Chicago, IL, USA) and Graphpad Prism version 6.04 (GraphPad Software, Inc., La Jolla, CA, USA). Normality was assessed using the Shapiro–Wilk test, histograms, and Q–Q plots. Normally distributed data are expressed as mean ± standard deviation and non-normally distributed data are expressed as median (interquartile range). Interstudy and inter-observer variability for subjects undergoing test-retest scans were compared using paired t-tests, co-efficients of variation and the Bland-Altmann method [28]. Inter-centre differences between subject demographics and native T1 values were assessed with independent sample t-tests or Mann-Whitney U tests for normally and non-normally distributed data respectively. Chi-squared tests and Fishers exact tests were used to assess for differences between nominal variables and are expressed as ‘count’ (%). Correlations between variables were assessed using Pearson’s and Spearman’s-rank analysis for normally and non-normally distributed data respectively.

### Sample size justification

A paired *t*-test sample size calculation was undertaken for the native T1 test-rest study, using the native T1 values from a previous study by our group conducted on the same 3 T MRI scanner (Skyra, Siemens Medical Imaging, Erlangen, Germany) [14]. To detect a 2.5% difference in native T1 times between test-retest scans (27 ms) with 90% power requires 8 patients to undergo test-retest scans. Ten patients were therefore recruited to undergo test-retest scans.

## Results

The demographic data for all patients from both centres are shown in Table 1. There were no statistically significant differences between the demographics of the groups. There was a trend towards longer dialysis vintage in participants from centre 1 compared to those in centre 2, but these difference did not reach statistical significance. There were no differences between the basal or the mid-ventricular native T1 times for HD patients between centre 1 and centre 2 respectively (basal T1 1280.5 ms ± 36.6 vs 1276.3 ms ± 32.3, *P* = 0.62 and mid T1 1282.6 ms ± 38.1 vs 1283.4 ± 39.3, *P* = 0.42). All scans were analysable and included in the analysis.

**Table 1:** Demographic details from both study centres

Variable	Centre 1 (*n* = 10)	Centre 2 (*n* = 10)
Age (Years)	57.8 ± 15	58 ± 13.5
Male (%)	8 (80%)	7 (70%)
HR (bpm)	76 ± 14	68 ± 11.6
SBP (mmHg)	143 ± 33	143 ± 19
DBP (mmHg)	80. ± 15	72 ± 13
Dialysis Vintage (Months)	26 ± 26.2	9 ± 3.9
Past Medical and Drug History		
HTN (n,%)	8 (80%)	6 (60%)
Diabetes (n,%)	3 (30%)	2 (20%)
Previous MI (n,%)	1 (10%)	2 (20%)
CAD (n,%)	3 (30%)	3 (30%)
PVD (n,%)	0 (0%)	1 (10%)
ACEi (n,%)	3 (30%)	1 (10%)
ARB (n%)	1 (10%)	0 (0%)
Diuretic (n,%)	1 (10%)	2 (20%)
Beta Blocker (n,%)*	4 (40%)	7 (70%)
Statin (n,%)	4 (40%)	5 (50%)
Calcium Channel Blocker (n,%)	3 (30%)	2 (20%)
Number of antihypertensives	0.96 ± 0.9	1.1 ± 0.9

Mean values with standard deviation expressed as n ± SD. N, % = Chi-squared + %. No significant differences were observed between any baseline demographic details

*bpm* beats per minute, *ACEi* angiotensin converting enzyme inhibitor, *ARB* angiotensin receptor blocker, *CAD* coronary artery disease, *DBP* diastolic blood pressure, *HR* heart rate, *HTN* hypertension, *MI* myocardial infarction, *PVD* peripheral vascular disease, *SBP* systolic blood pressure

*statistically significant difference between groups (P<0.05)

### Inter-study reproducibility of LV masses and volumes

**Fig. 2:**
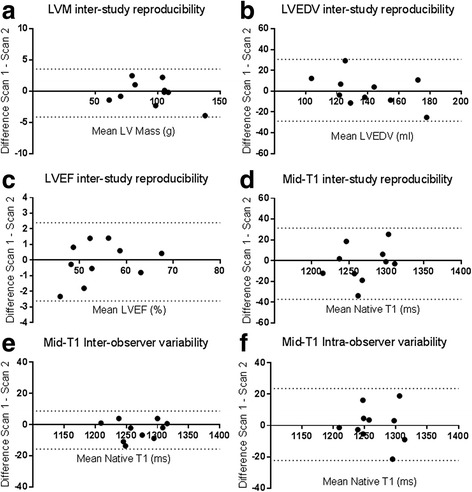
Bland-Altman plots for: **a**: Inter-study reproducibility left ventricular mass, **b**: Inter-study reproducibility left ventricular end-diastolic volume, **c**: Inter-study reproducibility left ventricular ejection fraction, **d**: Inter-study reproducibility mid-ventricular native T1, **e**: inter-observer variability of mid-ventricular native T1 and **f**: intra-observer variability of mid-ventricular native T1

**Fig. 3:**
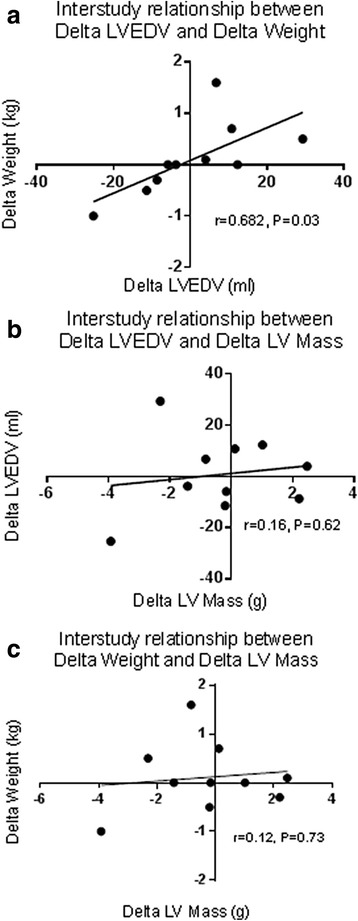
Relationships between measures of fluid status between scans and LV mass. **a**: Significant correlation between Delta LVEDV and Delta weight. **b**: No relationship between Delta LVEDV and Delta LV Mass or **c**: between Delta Weight and Delta LV mass. LV, left ventricular; LVEDV, Left ventricular end-diastolic volume

The mean interval between scans for patients undergoing test-retest inter-study reproducibility at centre 1 was 7 ± 4 days. Image quality was either good (*n* = 4) or excellent (*n* = 16) and of sufficient quality for quantitative analysis. The inter-study reproducibility, including coefficients of variation (CoV), bias and limits of agreement for LV mass, LVEDV, LVESV and LVEF are shown in Table 2. Bland-Altman plots did not show evidence of systematic bias, with all data points within 95% confidence intervals (Fig. 2). Interstudy mean difference and 95% confidence intervals of LVEDV and weight were 11.79 ml (5.6,18.0) and 0.5 kg (0.1,0.8) respectively. There was a significant correlation between change in LVEDV (ΔLVEDV) and change in weight (Δweight) between scans (*r* = 0.682, *P* = 0.03), but no association was found between change in LV mass (ΔLVMass) and ΔLVEDV or Δweight (*r* = 0.16, *P* = 0.67 and *r* = 0.12, *P* = 0.73 respectively) (Fig. 3).

**Table 2:** Interstudy reproducibility of LV mass, volumes and mid-ventricular native T1 values and Inter-observer and intra-observer variability of mid-ventricular native T1 values

Parameter	Study 1	Study 2	CoV	BIAS ± SD Difference	BA Limits of Agreement
Inter-study reproducibility					
LV Mass (diastolic) (g)	95.2 ± 22.0	95.5 ± 22.7	1.0%	-0.3 ± 1.96	-4.5–3.5
LVEDV (ml)	139.3 ± 21.0	138.5 ± 27.8	5.2%	0.8 ± 15.2	-28.9–30.5
LVESV (ml)	64.3 ± 16.3	64.2 ± 19.7	5.6%	0.1 ± 7.6	-14.8–15
LVEF (%)	54.3 ± 7.2	54.4 ± 6.8	1.1%	-0.1 ± 1.3	-2.6–2.4
Mid-ventricular native T1 (ms)	1267.8 ± 35.4	1270.7 ± 30.5	0.7%	-2.9 ± 17.5	-37.2–31.3
Inter-observer variability					
Mid-ventricular native T1 (ms)	1267.6 ± 35.4	1271 ± 34.8	0.3%	-3.4 ± 6.2	-15.6–8.8
Intra-observer variability					
Mid-ventricular native T1 (ms)	1267 ± 34.3	1266 ± 35.5	0.4%	0.66 ± 11.7	-22.3–23.7

Mean values with standard deviation expressed as n ± SD

*BA* Bland-Altman, *LV* left ventricular, *LVEDV* left ventricular end-diastolic volume, *LVESV* left ventricular end-systolic volume, *LVEF* left ventricular ejection fraction

### Inter-study reproducibility, inter-observer and intra-observer variability of mid-ventricular native T1

**Fig. 4:**
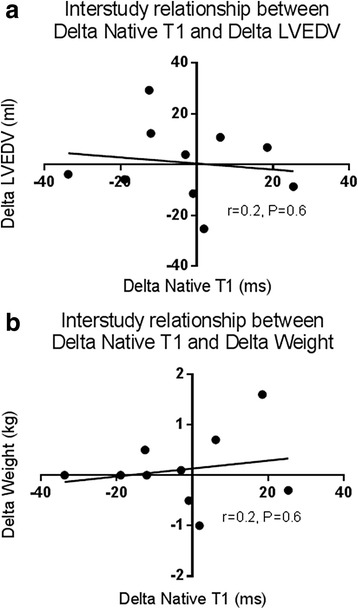
Relationships between changes in native T1 and changes in measures of fluid status. **a**: No correlation between delta native T1 and delta LVEDV. **b**: No relationship between delta native T1 and delta weight

Six out of 120 native T1 segments from mid-ventricular native T1 parametric maps were excluded from analysis due to artefact. The same segments were excluded from analysis by the two readers in the inter-observer analysis (one segment from interventricular septum and five from the lateral wall). The inter-study reproducibility, inter-observer and intra-observer variability of mid-ventricular native T1 values, including CoV, bias and limits of agreement for mid-LV native T1 values are shown in Table 2. Bland-Altman plots did not show evidence of any systematic bias, with all data points within 95% confidence intervals (Fig. 2). There was no significant correlation between change in mid-ventricular native T1 (ΔT1) or change in LVEDV (ΔLVEDV) and change in weight (Δweight) between scans (*r* = -0.14, *P* = 0.7 and *r* = 0.2, *P* = 0.6, respectively) (Fig. 4). Linear regression confirmed ΔT1 was unaffected by both ΔLVEDV and Δweight (adj *R*2 = 0.1, *P* = 0.71 and adj *R*2 = 0.08, *P* = 0.59). Based on these results, to detect a 2.5% difference in native T1 values with 90% power would require 13 patients and to detect a 5% difference in native T1 values with 90% power would require 5 patients (alpha error = 0.05 for both).

### Inter-centre measurement variability of basal and mid-ventricular native T1

Twenty-five out of 240 native T1 segments were excluded from analysis due to artefact (9 segments were excluded from the interventricular septum and 16 from the later wall). The two readers agreed on the analysability of 220/240 segments reviewed (91.7%). The inter-centre variability of native T1 analysis techniques of basal and mid-ventricular native T1 values, including CoV, bias and limits of agreement are shown in Table 3. Bland-Altman plots did not show evidence of systematic bias (Additional file 1: Appendix 1), although single data points fell just outside the 95% confidence intervals for mid-ventricular native T1 of centre 1 and for basal-ventricular and mid-ventricular native T1 for centre 2.

**Table 3:** Inter-centre variability of basal and mid-ventricular native T1 analysis techniques

Parameter	Centre 1 analysis	Centre 2 analysis	CoV	Bias ± SD Difference	BA Limits of Agreement
Centre 1 native T1 images					
Basal native T1 (ms)	1283.8 ± 38.7	1281.4 ± 39.6	0.8%	2.4 ± 21.5	-39.7–44.6
Mid native T1 (ms)	1281.3 ± 39.6	1279.8 ± 35.6	1.2%	1.5 ± 31.2	-59.7–62.6
Centre 2 native T1 images					
Basal native T1 (ms)	1290.4 ± 36.7	1276.4 ± 42.5	0.8%	14.1 ± 17.2	-19.7–47.8
Mid native T1 (ms)	1281.3 ± 31.6	1271.3 ± 33.9	0.9%	10 ± 23.1	-35–55.2

Mean values with standard deviation expressed as n ± SD

*BA* Bland-Altman

### T1 values of standard phantom between centres

Excellent agreement was seen for the phantom results obtained at both centres with no clinically relevant differences between scanners. Allowing for standard deviations, the phantom considered representative of a typical myocardial tissue T1 time, gave very similar T1 values at both sites (centre 1: 1192.7 ± 7.5 ms, centre 2: 1205.5 ± 5 ms). For the phantom blood pool T1 times obtained were again equivalent at each centre: (centre 1: 1899.2 ± 6.9 ms, centre 2: 1907.9 ± 10.7 ms).

## Discussion

In this study we have shown, for the first time in patients with ESRD on HD, that the reproducibility of native T1 values are excellent. The reproducibility values for T1 mapping using the MOLLI sequence compare favourably with those previously reported. Previous CoVs for inter-study reproducibility have been between 1.5 and 8.4%, [14–16, 29] compared with 0.7% in this report. As previously described, native T1 is related to water content of the relevant tissue [30] and the finding in this study that ΔT1 between test-re-test scans was unrelated to changes in fluid status suggests that changes in fluid status at the levels we have seen do not directly affect native T1 values in HD patients, although we accept that this study is only in 10 patients. Whilst the absolute changes in inter-study fluid status that we report are not large, they are the types of fluctuations in fluid you would expect to see in HD patients scanned on a non-dialysis day on week apart and therefore clinically relevant. This is a crucial observation in defining the reliability of T1 time as a potential measure of myocardial fibrosis in this patient group.

We have demonstrated that standardization of native T1 mapping analysis between centres is eminently possible. The agreement between the analysis techniques of centres is encouraging. Both techniques define segmental native T1 values for basal- and mid-ventricular slices as per the American Heart Association model (Fig. 1), with the average of these segments taken to be the mean native T1 for each slice. When areas of artefact are identified, affected segments can be excluded with the mean native T1 calculated from remaining unaffected segments. This way of assessing native T1 is likely to be more reproducible than alternative methods, where single regions of interest are drawn in the inter-ventricular septum [31]. Such methods are prone to sampling differences in native T1 times depending on where the region of interest is drawn. We have previously demonstrated that septal native T1 is significantly higher than non-septal native T1 in HD patients [18]. Moreover, a recent paper by Rauhalammi et al showed that native T1 values are significantly higher in the interventricular septum of health volunteer patients than non-septal myocardium [27] so analysis techniques that measure only the interventricular septal native T1 cannot be thought of as equivalent to mean circumferential ventricular native T1 values. The phantom analysis conducted at centre 1 and centre 2 confirmed the scanners and scan sequences at the different centres gave very similar results, with the differences in absolute values being clinically irrelevant. The mean basal- and mid-ventricular native T1 values between the two centres were virtually identical. Given that subjects from both centres were well matched in terms of demography and medical co-morbidity, it appears that the native T1 values generated at both centres are commensurate with each other. It should be noted that both centres used Siemens 3 T CMR scanners and whilst they were not the same model scanner, inter-vendor comparisons of myocardial native T1 values in HD patients are required. Ideally assessment of the reproducibility of native T1 times at different field strengths and between vendors and centres should also be carried out.

Although native T1 time has been shown to correlate well with histological levels of myocardial fibrosis in diseases of pressure overload [12] we still do not have histological confirmation that this is the case for HD patients; indeed low grade inflammation may increase native T1 [32]. Low grade inflammation is known to be a feature of CKD and for patients with ESRD on HD [33], so histological studies are required to confirm increased T1 times are due to myocardial fibrosis and not low grade inflammation or an additional disease process such as amyloidosis [34]. Whilst our results suggest inter-compartmental fluid shifts do not affect native T1 values, further, confirmatory studies should be considered, including studies that look at the acute effects of HD and ultrafiltration on native T1 values.

The inter-study reproducibility of traditional CMR measures of LV structure and function, including LV mass, LVEDV and LVEF were also excellent as has been shown previously for patients with ESRD [35]. The reproducibility of LV mass is particularly important as LV mass is known to be overestimated when imaged with echocardiography [36]. These findings confirm previous studies in HD patients showing conservation of measurement of LV mass with CMR in patients with differing LVEDV’s and fluid and loading status [35].

In 2011, Sado et al outlined a framework for development of imaging techniques to measure myocardial fibrosis, to ensure the robustness of the techniques [37]. They suggested that to be considered a reliable imaging biomarker of myocardial fibrosis, techniques should: detect changes in established disease states compared with controls; correlate with cardiac markers fibrosis (e.g., diastolic function, LVH); correlate with blood biomarkers of cardiac fibrosis; be able to track changes over time; be standardized in the way they are carried out (inter-vendor/inter-centre); be proven to compare closely with histological specimens from human subjects; and changes in the imaging biomarker should track changes in the disease after treatment. Work by our respective groups has already shown that native T1 values are higher than healthy and co-morbidity matched controls [17, 18]. Rutherford et al demonstrated that global native T1 values in HD correlate with LV mass indices (*r* = 0.452, *P* = 0.008), and that septal T1 values correlate with pre-dialysis high-sensitive troponin T (*r* = 0.397, *P* = 0.027) [17]. In addition to this, Graham-Brown et al showed that native T1 values correlate with measures of global circumferential and longitudinal strain (*r* = 0.41, *P* = 0.002, *r* = 0.55, *P* < 0.001) and septal native T1 correlated with septal systolic strain (*r* = 0.46, *P* < 0.001) [18].

Following the framework outlined by Sado et al, histological confirmation of the relationship between myocardial fibrosis and native T1 mapping now appears to be the final step required before longitudinal and interventional studies can be undertaken to define whether native T1 mapping is able to: track progression of myocardial fibrosis over time; assess how baseline or changes in native T1 values relate to outcomes; whether interventions that attempt to modify/improve fibrosis are measurable by native T1 mapping; and whether this modification improves outcomes for patients. The excellent reproducibility of native T1 mapping makes it a biomarker with great potential for the measurement of myocardial fibrosis in HD patients. Based on the results we have presented, only small numbers of patients would be required to adequately power interventional clinical trials that seek to reduce myocardial fibrosis. Whilst historically LV mass has been the surrogate end-point for clinical trial work that sought to improve CV outcomes, a recent systematic review and meta-analysis has called into question whether interventions that improve LV mass actually improve outcomes for patients with CKD and ESRD [5]. It should be noted that the neutral result of this meta-analysis might be due to inadequate power. Post-mortem histo-pathological studies have shown us that extent of myocardial fibrosis is the best predictor of outcome in patients with CKD and ESRD [6] and there is evidence in animal models, humans and even HD patients that regression of LVH is accompanied by reductions in measures of myocardial fibrosis [38–41]. Given that multiple studies have suggested that reducing LV mass improves outcomes for patients with ESRD [42], it could be that previous trials that have shown reductions in LV mass have actually just reflected a crude measure of a reduction in myocardial fibrosis content and this has led to the conflicting results about the importance of LV mass reduction and CV risk that has recently been reported. Given the central association between fibrosis, LVH, morbidity and mortality in HD patients, being able to reliably measure diffuse myocardial fibrosis is crucial to understanding its potential as a research and clinical end point.

## Conclusion

Native T1 mapping is an extremely reproducible technique in HD patients. Native T1 times seem to be unaffected by the changes in fluid status to which HD patients are prone. Further work is required to determine whether native myocardial T1 is related to prognosis in ESRD and whether interventions that reduce T1 are associated with improved outcomes.

## Additional file

Additional file 1: Appendix 1. Bland-Altman plots for variability of inter-centre analysis techniques of A: Basal-ventricular native T1 values of centre 1. B: Mid-ventricular native T1 values of centre 1. C: Basal-ventricular native T1 values of centre 2. D: Mid-ventricular native T1 values of centre 2. (TIF 574 kb)
