# Supplementary figures and images for: Native T1 mapping: inter-study, inter-observer and inter-center reproducibility in hemodialysis patients
Source: J Cardiovasc Magn Reson. 2017 Feb 27;19:21. doi: 10.1186/s12968-017-0337-7 (PMC5327541; doi:10.1186/s12968-017-0337-7)

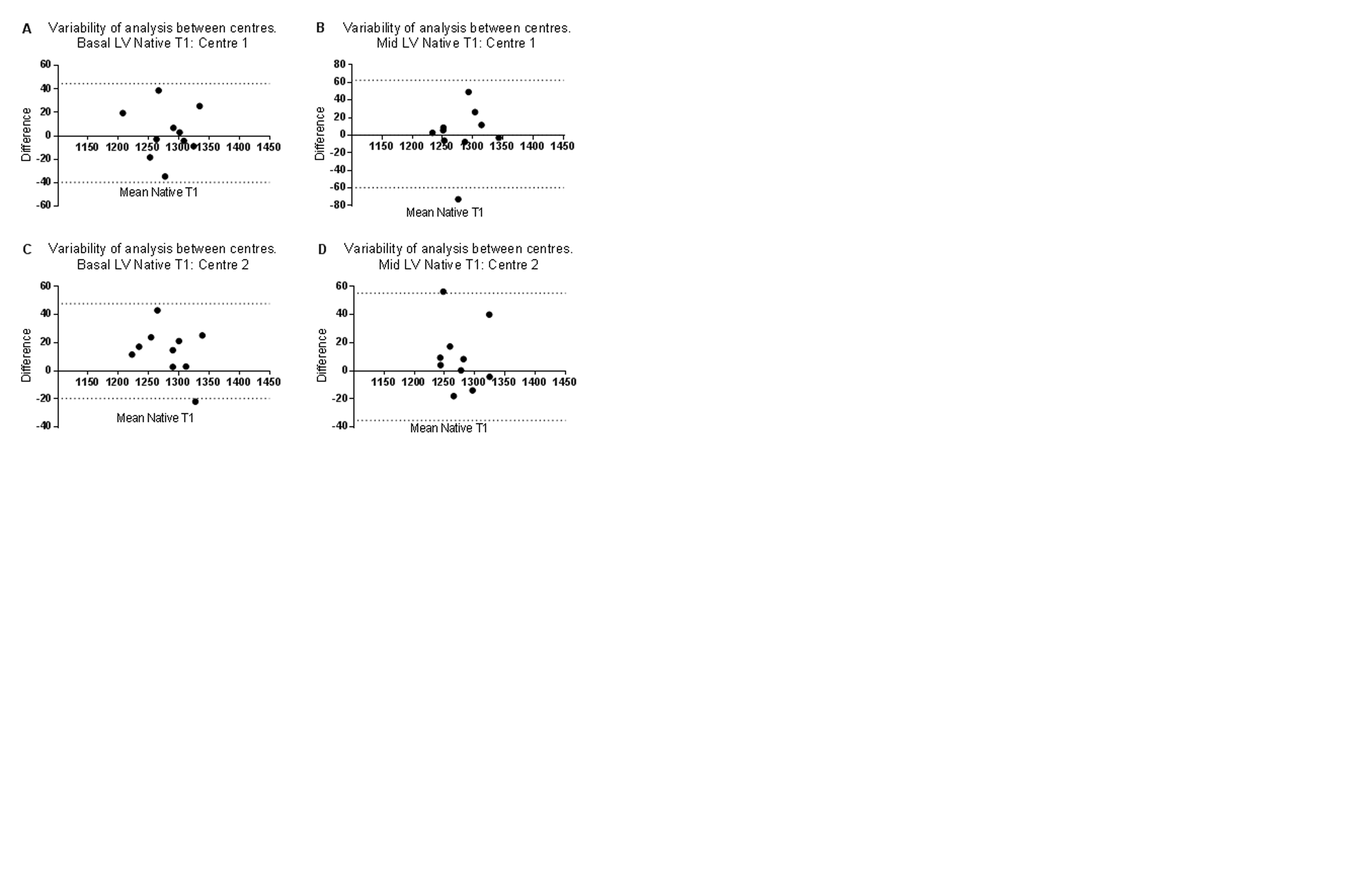

Supplement: Additional file 1: Appendix 1. — Bland-Altman plots for variability of inter-centre analysis techniques of A: Basal-ventricular native T1 values of centre 1. B: Mid-ventricular native T1 values of centre 1. C: Basal-ventricular native T1 values of centre 2. D: Mid-ventricular native T1 values of centre 2. (TIF 574 kb) [file 12968_2017_337_MOESM1_ESM.tif]
